# Lactic Acid Bacteria Postbiotics as Adjunctives to Glioblastoma Therapy to Fight Treatment Escape and Protect Non-Neoplastic Cells from Side Effects

**DOI:** 10.3390/cells15030226

**Published:** 2026-01-24

**Authors:** Pola Głowacka, Agnieszka Pudlarz, Joanna Wasiak, Magdalena Peszyńska-Piorun, Michał Biegała, Karol Wiśniewski, Dariusz J. Jaskólski, Adam Marek Pieczonka, Tomasz Płoszaj, Janusz Szemraj, Monika Witusik-Perkowska

**Affiliations:** 1 Department of Medical Biochemistry, Medical University of Lodz, 6/8 Mazowiecka Str., 92-215 Lodz, Poland; pola.glowacka@umed.lodz.pl (P.G.);; 2 International Doctoral School, Medical University of Lodz, 90-419 Lodz, Poland; 3 Radiotherapy Planning Department, Copernicus Memorial Hospital in Lodz Comprehensive Cancer Center and Traumatology, 93-513 Lodz, Poland; 4 Department of Medical Imaging Technology, Faculty of Medicine, Medical University of Lodz, Narutowicza 60, 90-136 Lodz, Poland; 5 Department of Medical Physics, Copernicus Memorial Hospital in Lodz Comprehensive Cancer Center and Traumatology, 93-513 Lodz, Poland; 6 Department of Neurosurgery and Neuro-Oncology, Medical University of Lodz, Barlicki University Hospital, Kopcinskiego St. 22, 90-153 Lodz, Poland; 7 Department of Organic and Applied Chemistry, Faculty of Chemistry, University of Lodz, Tamka 12, 91-403 Lodz, Poland; 8 Department of Clinical Genetics, Medical University of Lodz, Pomorska Str. 251, 92-213 Lodz, Poland

**Keywords:** postbiotics, glioblastoma, chemotherapy, radiotherapy, senescence, lactic acid bacteria

## Abstract

Despite tremendous scientific efforts aimed at glioblastoma’s (GB) ability to escape therapeutic attempts, the concern remains unsolved. Postbiotics, metabolites, and macromolecules of probiotic bacteria could become adjuvant therapeutics both dealing with cellular events constituting tumor therapy escape mechanisms and protecting normal cells from therapy-induced damage. The study aims to evaluate the dual potential of postbiotics obtained from lactic acid bacteria, *L. plantarum* and *L. rhamnosus*, on patient-derived and commercially available GB and normal cells alone and in combination with chemotherapeutic and irradiation oncotreatment regimens. Postbiotic mixtures (PMs) show cytoprotective potential against a new anti-cancer agent—ARA12—on astrocytes and cytoprotective action to irradiated normal fibroblast cells. Although GB cells’ apoptotic response varied between patient-derived cells, both PMs exert cytotoxic or cytostatic effects alone and, in most of the studied therapeutic combinations, on all tested GB cell lines. In particular, *L. plantarum* PM alleviates treatment escape, possibly shifting the tumor drug response from senescence to apoptosis. The results suggest that postbiotic-based adjunctive treatment could potentiate the therapeutic effect toward neoplastic cells, while alleviating chemotherapy’s adverse effects, helping clinicians to tackle the issue of therapy resistance and improve patients’ comfort.

## 1. Introduction

Glioblastoma (GB) is considered one of the most common and fatal brain neoplasms in adults. It originates from glial cells and is characterized by enormous intra-tumor molecular and cellular heterogeneity, both in terms of cells composition, their phenotype, and cellular states, which undergo dynamic changes, making the neoplasm extremely diverse both intra- and interpersonally [1]. Considering the genetic landscape of glioma, the mutational status of the isocitrate dehydrogenase (*IDH*) gene has been included in the WHO’s diagnostic criteria since 2021 as a clinically relevant factor, for it has been shown that IDH-wildtype tumors are more resistant to therapy [2]. The present study is based on GB cells harboring no *IDH* mutations.

Current therapy is based on maximal tumor resection, followed by irradiation and temozolomide-based chemotherapy; however, the malignancy remains practically incurable, as such a therapeutic regime results in a mere 14.6 months survival rate [3]. The bleak prognosis is widely ascribed to the mentioned tumor heterogeneity, its infiltrative nature, and the blood–brain barrier limiting drug access to the tumor. Recently it has been discussed that the present therapies are focused on GB cells with insufficient attention paid to the tumor microenvironment and treatment-induced undesired effects that paradoxically lead to acquired resistance [4,5,6,7].

Moreover, standard chemotherapeutics, including temozolomide, induce neuroinflammation and cellular senescence, leading to tumor therapy escape mechanisms in neoplastic cells and harmful changes in normal cells, resulting in the accumulation of senescent cells that has been linked to various disorders including neurodegeneration. Therapy-induced senescence poses a serious challenge in glioblastoma treatment, as the recurrence of the tumor may be ascribed to the senescent cells, aggravating inflammatory processes [8,9,10,11].

The dismal prognoses for GB and the extent of normal tissue damage caused by applied therapeutics still drive scientists’ search for effective radio- and chemo-sensitizers, as well as other adjuvants that could allow for limiting the bystander effect on normal cells while effectively targeting the tumor and its tumorigenic environment [12].

The current study includes an examination of a new drug candidate, an aziridine–hydrazide hydrazone derivative—ARA12—whose high proapoptotic potential has already been proven on GB cells and breast cancer cells [13,14], as well as postbiotics, a vast group of various probiotic bacteria metabolites, not viable bacteria, and their fragments that show beneficial activities towards the host’s health and well-being. Their numerous healthful properties include bacteriostatic activity towards pathogenic microorganisms, antioxidant activity, and cytoprotective properties to normal cells while being selectively cytotoxic to neoplastic ones; they were also observed to influence immunoregulation and ameliorate various gastrointestinal disorders [15,16,17]. The metabolites include short-chain fatty acids (SCFAs), nucleic and other organic acids, polysaccharides, enzymes, phenolic compounds, polyamines, and extracellular vesicles, plenty of which are proven to pass the blood–brain barrier, and therefore affect not only the intestines and organs directly supplied by systemic circulation, but also readily interact with brain cells [18].

Among others, postbiotics were observed to exert anti-cancer influence on colorectal cancer, gastric cancer, breast cancer, and cervical cancer cells as well as on leukemia and hepatocellular carcinoma in vitro models [19,20,21]. Postbiotic-based adjunctive anti-cancer therapy has been tested on breast cancer cells and myeloid cells [14,22]. However, studies elucidating the influence of postbiotics on brain tumors, including GB cells, are still scarce.

Patients diagnosed with glioblastoma have been reported to suffer major microbiota alterations, causing dysbiosis that induces a shortage of beneficial bacterial metabolites, such as short-chain fatty acids (SCFAs),. Such shortage can negatively affect patients’ mood and can further aggravate the already increased permeability of the blood–brain barrier [23]. More studies regarding the relationship between the microbiome of GB patients’ receiving chemo-radiation and the therapeutic outcome and tumor’s recurrence rate are underway.

In the current study, we aim to deliver exploratory results regarding the influence exerted by postbiotics derived from lactic acid probiotic bacteria, *Lacticaseibacillus rhamnosus* and *Lactiplantibacillus plantarum*, on different GB cell lines: one commercial and four patient-derived (IDH-wildtype), as well as normal human cells. The selected LAB strains are renowned for their versatile health-promoting effects and proven safety, making them valuable components of probiotic supplements and medications on the market. *L. rhamnosus* was observed to produce butyrate, a SCFA with anti-neoplastic potential noted in plenty of research, while *L. plantarum* was implemented in lessening systemic inflammation and improving protein metabolism in cancer patients undergoing chemo- and radiotherapy. Postbiotics derived from both of these species have exerted beneficial therapeutic effects alone and in combination with routinely applied chemotherapeutics and the new drug candidate ARA12 on breast cancer cell line MCF7 [14,24,25,26].

Then, we verify if postbiotics sensitize neoplastic cells to chemotherapeutics: a standard anti-neoplastic drug–TMZ—and ARA12 (a compound examined previously as a new anti-neoplastic drug candidate), and if the same postbiotic mixture provides cytoprotection for normal cells. Lastly, we peruse the radio-sensitizing properties of postbiotics towards tumor cells and cytoprotective action towards normal cells.

## 2. Materials and Methods

### 2.1. Culturing Bacteria and Preparing Cell-Free Supernatant

Two strains of lactic acid bacteria, *Lacticaseibacillus rhamnosus GG* and *Lactiplantibacillus plantarum 299v*, were separately cultured in De Man, Rogosa, and Sharpe medium (MRS) at 30 °C with periodic shaking overnight with limited oxygen conditions. Then, a bacterial suspension with optical density (OD = 0.8) measured at 600 nm matching 10^8^ CFU/mL was obtained and the bacteria were transferred into antibiotic free cell culture media with FBS for overnight incubation at 30 °C for the purpose of eliminating the direct influence of MRS applied to cells. After incubation, bacteria and their metabolites suspended in DMEM underwent 20 min centrifugation at 4000× *g*, and after discarding the pellet, the obtained supernatants were adjusted to pH 7.4. To remove the remaining bacterial fragments, cell-free mixtures were filtered under sterile conditions through a 0.22 μm membrane and eventually utilized as a source of postbiotic metabolites, referred to as a postbiotic mixture (PM).

### 2.2. Cell Culture

Commercially available cells used as the GB model were the U87MG cell line (ATCC HTB-14, Manassas, MA, USA), and normal human astrocytes (NHAs; Lonza, Basel, Switzerland) as well as human diploid fibroblasts (WI-38; ATCC No. CCL-75, Manassas, MA, USA) were utilized as normal control cells. The NHA cell line was cultured accordingly with the manufacturer’s protocol (Lonza, Basel, Switzerland) while U87MG and fibroblasts were cultured in Dulbecco’s modified Eagle’s medium, respectively, DMEM/F12 and DMEM—high glucose (DMEM/F12, Gibco and DMEM-HG, Gibco, Life Technologies Europe B.V., Bleiswijk, The Netherlands)—with 10% FBS (Gibco, Life Technologies Limited, Paisley, UK) and the antibiotics gentamicin (100 mg/mL) and streptomycin with penicillin (100 mg/mL) (Sigma-Aldrich, St. Louis, MO, USA). All mentioned cell cultures were conducted at 37 °C and 5% CO_2_ atmosphere.

Glioblastoma (IDH-wildtype) samples, verified according to the WHO criteria [2], devoted for cell culture establishment were obtained from the Department of Neurosurgery and Oncology of the Central Nervous System, Medical University of Lodz, Poland. Informed consent was obtained from all the patients, in compliance with the Declaration of Helsinki, and the procedures were performed following the ethical standards of the Bioethics Committee of the Medical University of Lodz (Approval Ref. No. RNN/211/23/KE). Glioblastoma primary cultures were derived from 4 tumor samples according to the procedure of culture generation described in our previous reports [27,28]. Subsequently, the GB cells were cultured as an adherent model in serum-supplemented medium (DMEM/F12, Gibco, Life Technologies Europe B.V., Bleiswijk, The Netherlands; with 10% FBS, Gibco, Life Technologies Limited, Paisley, UK; and antibiotics: Gentamycin solution and Penicilin-Streptomycin, Sigma-Aldrich, St. Louis, MO, USA). The cells were passaged and expanded following every 3–5 days (depending on proliferation activity). The neoplastic cells’ statuses were verified based on the expression of astrocytoma-associated antigens IL13Rα2, Fra-1, and EphA2, as previously described [27]. Due to the GB intra-heterogeneity and the possibility of clone selection in culture, *IDH* 1/2 unmutated status in cultivated cell cultures was confirmed by real-time polymerase chain reaction for two of the most clinically relevant codons: *IDH*1 R132H and *IDH*2 R172K [29,30].

### 2.3. Compounds: Natural and Synthetic

*L. plantarum 299v* or *L. rhamnosus GG* postbiotics were obtained as described above. TMZ (PHR1437, Sigma Aldrich) and ARA12, a novel derivative of aziridine–hydrazide hydrazones, was synthesized in accordance with the protocol previously presented [13].

### 2.4. IDH Status Verification

In order to verify the status of *IDH*1/2 in cultivated GB cells, EasyPGX^®^ready IDH1/2 kit (Diatech Pharmacogenetics, Jesi, Italy) was utilized for mutations of crucial clinical importance: *IDH*1 R132H and *IDH*2 R172K. The DNA from the cell culture was extracted using a Genomic Mini kit (A&A Biotechnology, Gdynia, Poland). The real-time PCR was carried out accordingly with EasyPGX^®^ready IDH1/2 kit’s manufacturer protocol for *IDH*1 R132H and *IDH*2 R172K mutations.

### 2.5. Cell Viability Assay

The GB cells (U87MG line and patient derived cultures) and NHA and WI-38 were seeded into 96-well microplates at 5 × 10^3^/well and incubated at 37 °C in a 5% CO_2_ for 24 h, then the cells were treated with the examined compounds—the drug TMZ (500 µM) and drug candidate ARA12 (50 μg/mL)—as well as postbiotics, alone or in combination as drug–postbiotic.

Firstly, to establish the most beneficial concentration of PM from *L. plantarum* or *L. rhamnosus*, U87MG cells were subjected to PM concentrations ranging from 0 to 40% (*v*/*v* of cell culture medium). The *L. plantarum* or *L. rhamnsous* PM concentration selected for the following analyses was 30% (*v*/*v*). It was then applied to NHA and WI-38 cells to assess their influence on non-neoplastic cells.

The selection of concentrations of synthetic compounds was based on previously described examinations [13]. The usual incubation time for drug treatment was 72 h unless stated otherwise.

For irradiation treatment, cells were planted in the 96-well plates as previously described; the PM was added after 24 h and then the cells were irradiated with 2, 4, 8, and 12 Gy after 24 h. The following analyses were conducted after 72 h incubation.

To assess cell viability in the abovementioned experiments, the PrestoBlue™ Cell Viability Reagent (Thermo Scientific, Rockford, IL, USA) was applied accordingly with the manufacturer’s protocol. A microplate reader (Glomax Multi Detection System; Promega, Madison, WI, USA) was used to record the fluorescence signal.

### 2.6. Cell Death Assay—Annexin V/PI Staining

For apoptosis detection in GB cell lines, the Annexin V Apoptosis Detection Kit (BD Biosciences, Erembodegem, Belgium) was utilized. After seeding GB cells into 6-well plates at 1 × 10^5^/well, 24 h incubation at 37 °C in 5% CO_2_ followed. Then, cells were incubated for 72 h with postbiotics or the examined drugs alone—30% (*v*/*v*) postbiotics, TMZ (500 µM), and ARA12 (50 μg/mL)—or in combination with PMs from *L. plantarum* and *L. rhamnosus* (30% (*v*/*v*)). For irradiation, plated cells were pretreated with PM for 24 h, then irradiated with 12 Gy and incubated for 72. After incubation, cell pellets were suspended in PBS, centrifuged, and stained with annexin V-FITC/propidium iodide (PI) at room temperature in the dark for 15 min, as advised by the manufacturer. Flow cytometry cellular analyses were carried out on CytoFLEX Beckman Coulter (Brea, CA, USA).

### 2.7. Proliferation Analysis

To assess the influence of particular treatment modes on the proliferation status of GB cells, the Click-iT^®^ EdU Alexa Fluor^®^ 488 Imaging Kit (Invitrogen, Waltham, MA, USA) was utilized.

Cells were seeded in 4-well plates at 2 × 10^4^/well and incubated at 37 °C in 5% CO_2_. After 24 h, the cells were treated with postbiotics or the examined drugs alone—30% of postbiotic mixture of cell culture media (30% *v*/*v*), TMZ (500 µM), and ARA12 (50 μg/mL)—or in combination with PMs from *L. plantarum* and *L. rhamnosus* in a concentration of 30% (*v*/*v*) for 48 h. For irradiation, plated cells were pretreated with PM for 24 h, then irradiated with 8 Gy and incubated for 72 h. Then, EdU (5-ethynyl-2′-deoxyuridine) assay was performed according to the instructions of the manufacturer. Briefly, cells were incubated for 2.5 h with the EdU component at 10 µM, then fixed with paraformaldehyde and permeabilized with Triton^®^ X-100 (Thermo Fisher Scientific, Waltham, MA, USA), washed and incubated for 30 min with Click-iT^®^ reaction cocktail, then washed and mounted with ProLong Glass Antifade Moutant with NucBlue Stain (Invitrogen, P36981, Waltham, MA, USA), and then analyzed with fluorescent microscopy. Cells were counted with Fiji version 2.16.0.

### 2.8. SA-β-Gal Detection-Based Cellular Senescence Assay

Cells were seeded in 4-well plates at 2 × 10^4^/well and incubated at 37 °C in 5% CO_2_. After 24 h, the cells were treated with postbiotics or the examined drugs alone—30% (*v*/*v*) postbiotics, TMZ (500 µM), and ARA12 (50 μg/mL)—or in combination with bacterial supernatants from *L. plantarum* and *L. rhamnosus* in a concentration of 30% (*v*/*v*) and incubated for 72 h for TMZ and 24 h for ARA12. For irradiation experiments, cells were seeded and treated with bacterial supernatants after 24 h and irradiated after another 24 h and incubated for 72 h. Then, cells were subjected to the cellular senescence assay (KAA002, Sigma-Aldrich, Burlington, MA, USA), according to the manufacturer’s instructions. Briefly, cells were fixed and stained overnight with SA-β-gal (senescence-associated beta-galactosidase) Detection Solution, then washed and observed under light microscope. Due to the specificity of glioblastoma growth and its affinity to form clusters, a semiquantitative analysis was performed based on the assessment of percentage of the area occupied by cells positive for SA-β-gal. For each experimental condition with no less than 100 cells from 4 separate microphotographs from independent experiments was counted. The results were obtained with Fiji version 2.16.0.

### 2.9. Statistical Analysis

Statistical analyses were performed in GraphPad Prism 8.2.1. For determining differences among three or more groups, one-way or two-way analysis of variance (ANOVA) was used, as was applicable for a particular case, followed by a post hoc test (Holms–Sidak) when appropriate. The value of *p* < 0.05 was considered statistically significant.

## 3. Results

### 3.1. Initial Screening of L. plantarum and L. rhamnosus-Derived PM Activity Shows Cytotoxicity Towards GB Cells and Cytoprotective Potential Against Anti-Cancer Agent—ARA12

**Figure 1:**
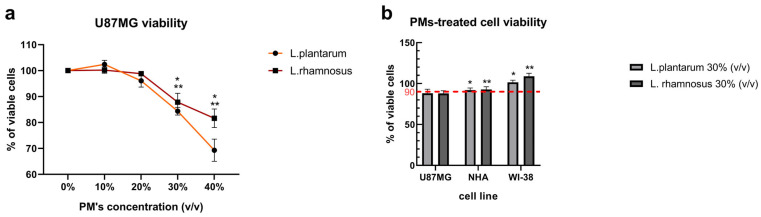
Concentration-dependent influence of PMs on viability of GB and non-neoplastic cell lines. (**a**) The response of U87MG cells to 72 h incubation with increasing concentrations (*v*/*v*) of *L. plantarum*- and *L. rhamnosus*-derived PMs based on resazurin viability assay PrestoBlue. Statistically significant differences in cell viability compared to untreated control are indicated by * for PM derived from *L. plantarum* and ** for *L. rhamnosus*. (**b**) The response of the U87MG GB cell line and normal cell lines: NHA and WI-38 treated with 30% PM (*v*/*v*) derived from *L. plantarum* and *L. rhamnosus* for 72 h. Viability was assessed by PrestoBlue assay and the presented percentage viability is normalized to the untreated control for each cell line. Statistically significant differences between U87MG and normal cell lines’ responses to PM application were marked with * for *L. plantarum* and ** for *L. rhamnosus*; (*p* < 0.05). PM—postbiotic mixture.

To evaluate and determine the best concentration of PMs applied in further experiments, increasing concentrations of PMs were applied to the GB in vitro model—the U87MG cell line. The results are depicted in Figure 1a. For 72 h incubation, the lowest PM concentration to result in a significant difference in the cells’ viability was 30% (*v*/*v*) of PM in cell culture medium—DMEM/F12—which decreased viability to 88.08% for *L. plantarum*-derived PM and 87.9% for *L. rhamnosus*-derived PM. Then, the PM concentration of 30% was tested on normal cell lines: normal human astrocytes (NHAs) and human diploid fibroblast cell line—WI-38—to verify the PMs’ selective cytotoxic activity towards neoplastic cells.

The results (Figure 1b) presented different sensitivities of normal cell lines to PMs, showing no cytotoxicity toward WI-38 and slight influence on NHA viability when compared to the untreated control (the percentage viability was normalized to the untreated control sample). Since the viability of normal cells did not fall below 90% of the untreated sample viability (91.26% ± 2.01% for *L. plantarum*-derived PM and 93.2% ± 3.1% for *L. rhamnosus*-derived PM for NHA cells and 101.65% ± 3.6% and 111.93% ± 1.76% for WI-38, respectively), and differences between the cytotoxic effect of both PMs on GB and normal cell lines were shown, as for U87MG cells, *L. plantarum* PM decreased cell viability to 87.45% ±1.48% while *L. rhamnosus* PM lowered it to 85.76% ± 4.3%, the concentration of 30% PM of cell culture medium (*v*/*v*) was selected for both PMs to be utilized in consecutive experiments.

**Figure 2:**
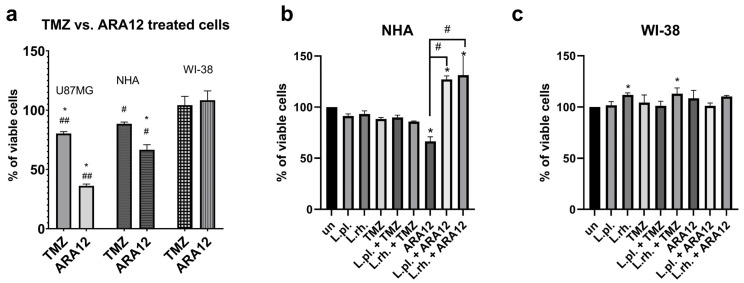
Cytoprotective potential of PM against anti-cancer compounds TMZ and ARA12. Analysis of cells’ viability by PrestoBlue assay in (**a**) a comparison of U87MG, NHA, and WI-38 treated with TMZ and ARA12 for 72 h; (**b**) NHA and (**c**) WI-38 cells in response to 72 h treatment with postbiotics with anti-neoplastic drugs: TMZ and ARA12. Statistically significant differences between samples are indicated with * when compared to untreated control, whereas significant differences between treatment modes on NHA cells are marked with # and ## on U87MG (*p* < 0.05). TMZ—temozolomide, L.pl.—*L. plantarum*, L.rh.—*L. rhamnosus*, un—untreated control.

Our previous report revealed promising anti-neoplastic activity of ARA12 against GB cells, stronger than temozolomide activity, but with limited selectivity in relation to normal cells (NHA) (Figure 2a) [12]. To evaluate the cytoprotective potential of LAB-derived PM, the non-cancerous cell lines (NHA, Figure 2b, and Wi-38, Figure 2c) were treated with a combination of synthetic anti-cancer agents (TMZ and ARA12) and PM.

Based on previous research, the applied concentration for TMZ was 500 µM. To minimize harmful effects on normal cells, ARA12 was applied in the lower concentration of 50 μg/mL, as sufficient to negatively influence the viability of GB cells [12]. NHA and WI-38 cells were treated with PMs and drugs alone and their combinations for 72 h. Although it did not significantly decrease the fibroblasts’ viability, the chemical was also more harmful towards NHA (66.56% of viable cells, *p* < 0.05), but to a lesser extent than towards U87MG. Moreover, both PMs had a considerable cytoprotective effect on NHA when applied with ARA12, boosting the viable cells’ percentages to 127.2% for *L. plantarum* PM and 131.29% for *L. rhamnosus* PM addition (<0.0001).

### 3.2. The Enhanced Proapoptotic, Antiproliferative, and Anti-Senescent Effect of L. plantarum- and L. rhamnosus-Derived PM on U87MG Glioblastoma Cell Line

To assess the anti-neoplastic activity of LAB-derived PM as potential adjunctive agents in GB therapy, we tested their influence on U87MG cell death, proliferation ability, and senescence in the presence of routine therapeutic TMZ and new anti-cancer candidate ARA12.

**Figure 3:**
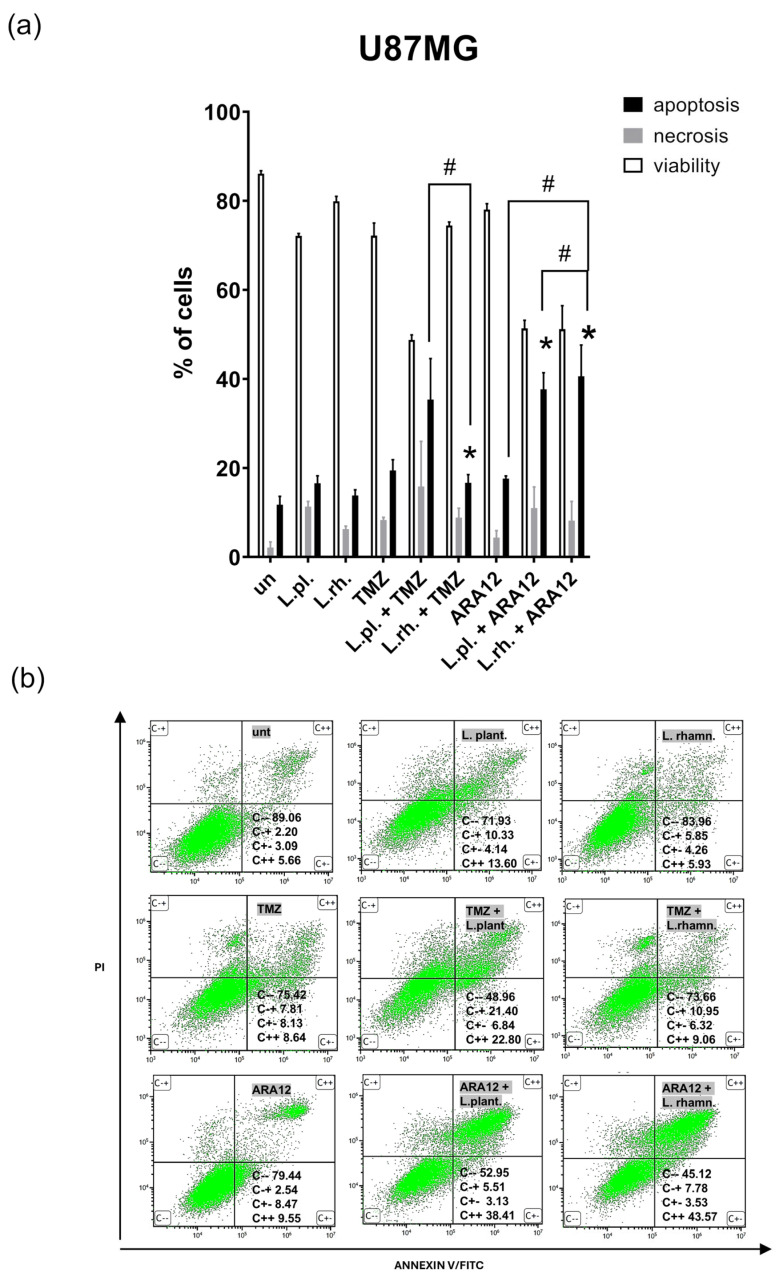
Cell death processes in U87MG cells treated with PMs and examined drugs. Analysis of cell death processes in U87MG cell line in response to 72 h treatment with postbiotics alone and their synergy with anti-neoplastic drugs. (**a**) The quantitative analysis of FC output of apoptosis assay and (**b**) the representative results of FC data (annexin V-FITC/PI staining). Statistically significant differences assessed for percentage of apoptotic cells between given sample and untreated cells are indicated with *, differences between samples are indicated with # (*p* < 0.05). C − − viable cells, C − + necrotic cells, C + − early apoptotic cells, C + + late apoptotic cells, TMZ—temozolomide, L.pl.—*L. plantarum*, L.rh.—*L. rhamnosus*, un—untreated control.

The results, presented in Figure 3, concerning cell death processes show that U87MG cells were sensitized by *L. plantarum*-derived PM to TMZ and by both PMs to ARA12, resulting in a significant increase in apoptotic cell population (19.46% of apoptotic cells for TMZ vs. 35.36% for TMZ with *L. plantarum* PM addition, *p* = 0.005, 17.6% for ARA12 alone vs. 36.38% for *L. plantarum* PM, *p* = 0.0005, and 40.6% for *L. rhamnosus* PM addition, *p* = 0.0001).

**Figure 4:**
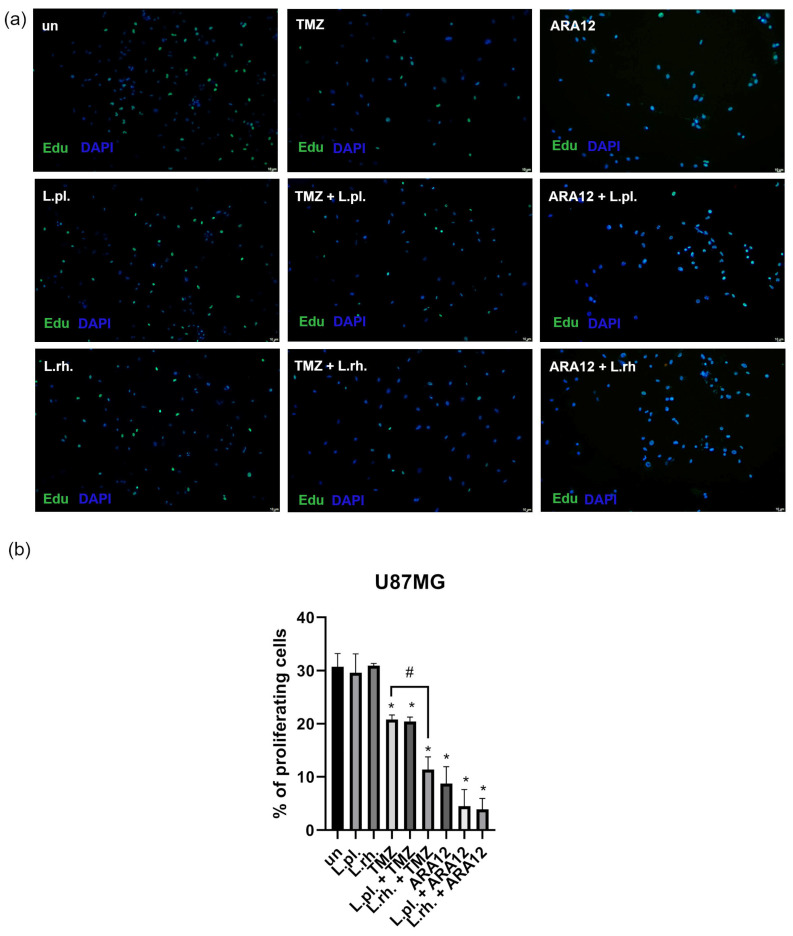
Proliferation rate changes in U87MG cells treated with examined compounds. U87MG cells’ proliferation rate in response to treatment with LAB-derived PM and their combinations with tested drugs for 48 h: (**a**) Representative results, (**b**) quantitative analysis. Statistically significant differences between given sample and untreated cells are indicated with *, differences between samples are indicated with # (*p* < 0.05). TMZ—temozolomide, L.pl.—*L. plantarum*, L.rh.—*L. rhamnosus*, un—untreated control.

In order to assess the cytostatic effect of PMs and their combinations with TMZ and ARA12 on the U87MG cell line, EdU-based proliferation assay was applied. The results are shown in Figure 4. The PMs alone did not significantly affect the proliferation rate;; however, *L. rhamnosus*-derived PM addition to TMZ resulted in a noticeable proliferation decline compared to TMZ alone (20.79% of cells were proliferating in the TMZ alone sample vs. 11.37% in the combined treatment condition, *p* = 0.0015). All treatment modes utilizing TMZ and ARA12 caused a significant proliferation decrease (*p* < 0.05); however, the cytostatic effect of ARA12, both alone and in combination, was more pronounced than that of TMZ (20.79% of EdU-positive cells (EdU+ cells) for TMZ, 20.42% for TMZ with *L. plantarum* PM, and 11.37% for TMZ with *L. rhamnosus* vs. 8.75% of EdU+ cells for ARA12, 4.51% for *L. plantarum* PM with ARA12, and 3.92% for *L. rhamnosus* PM with ARA12; *p* = 0.0001 between TMZ and ARA12). Moreover, when applying ARA12 with LAB-derived PMs, although statistically insignificant, the trend to enhance proliferation inhibition was also observed; however, the strong effect observed for ARA12 alone makes the differences difficult to determine, since only less than 10% of proliferating cells was detected for those treatment modes (ARA12, ARA12 + *L. plant.*, ARA12 + *L. rhamn.*).

**Figure 5:**
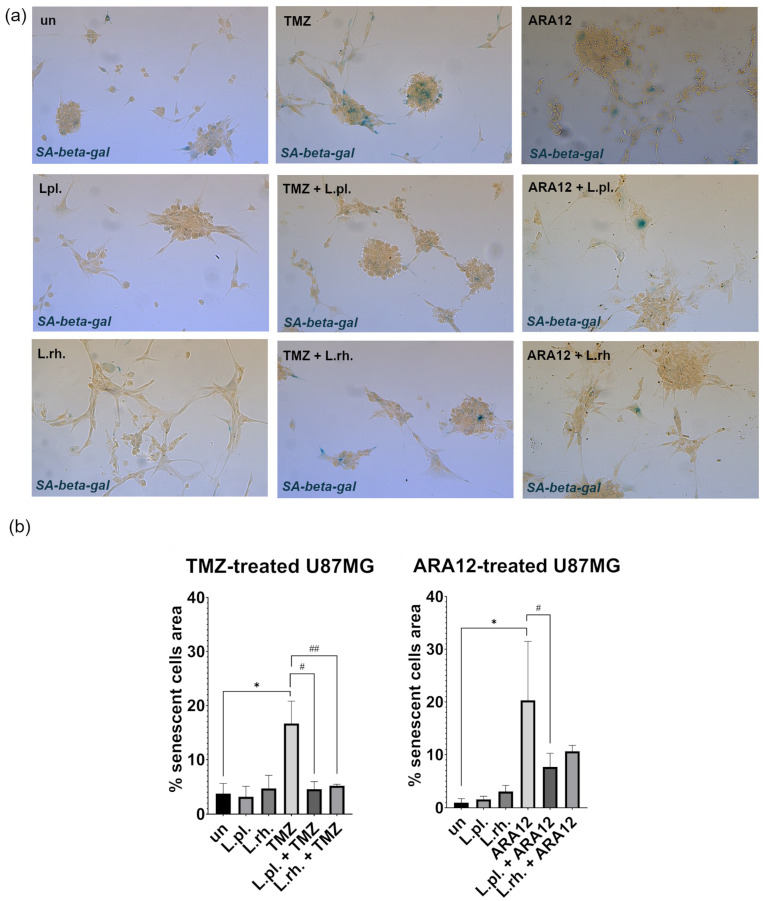
Senescence examination on PM- and drug-treated U87MG cells. SA-β-gal detection-based senescence assessment on U87MG cells in response to treatment with LAB-derived PM and their combinations with tested drugs. (**a**) Representative results of SA-β-gal assay (20x magnification) (**b**) Quantitative analysis of senescence assay for U87MG cells treated with TMZ (72 h incubation) and ARA12 (24 h incubation). Statistically significant differences between given sample and untreated cells are indicated with *, differences between samples are indicated with # for *L. plantarum* and ## for *L. rhamnosus* (*p* < 0.05). Due to the specificity of cell growth, forming clusters, the values are expressed as percentage of the positive signal area to the area occupied by cells. TMZ—temozolomide, L.pl.—*L. plantarum*, L.rh.—*L. rhamnosus*, un—untreated control, SA-β-gal—(senescence-associated beta-galactosidase).

Senescence assessment with SA-β-gal detection-based assay, performed with PMs and their combinations with TMZ and ARA12 (Figure 5), has revealed a pro-senescent effect of anti-neoplastic drugs, which is more pronounced in the case of drug candidate ARA12 (a nearly 20% increase compared to untreated cells) than TMZ (about 13% increase). Senescence was less pronounced in the case of both PMs combined with TMZ than in TMZ treatment alone (4.59% for *L. plantarum* combination and 5.23% for *L. rhamnosus* combination vs. 16.69% for TMZ alone, *p* < 0.0001). *L. plantarum*-derived PM was also observed to alleviate ARA12-induced senescence when applied in combination with the drug (7.7% for *L. plantarum* combination vs. 20.31% for ARA12 alone, *p* = 0.017). What is important, parallelly to senescence alleviation caused by combinatory treatment (TMZ and PMs, ARA12 and PMs), is that the enhancement of apoptosis was observed in comparison to U87MG cells treated with TMZ or ARA12 alone (Figure 3).

### 3.3. Patient-Derived GB Cell Lines Present Heterogenous Responsiveness to Proapoptotic and Antiproliferative Effect of L. plantarum- and L. rhamnosus-Derived PM in Combination with Anti-Neoplastic Drugs

**Figure 6:**
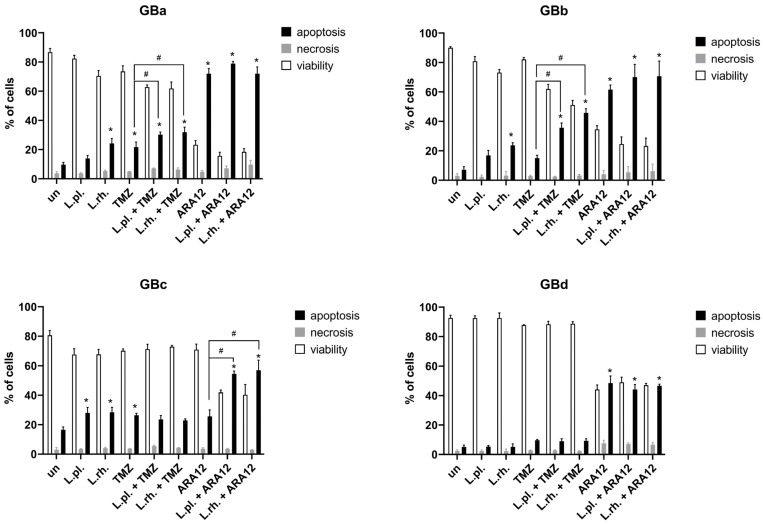
Analysis of cell death processes triggered by PMs and drugs in patient-derived GB cell lines. GBa, GBb, GBc, and GBd response to 72 h treatment with postbiotics alone and their combination with anti-neoplastic drugs. The results of FC analysis of apoptosis assay (annexin V/PI staining). Statistically significant differences assessed for percentage of apoptotic cells between given sample and untreated cells are indicated with *, differences between samples are indicated with #; (*p* < 0.05). TMZ—temozolomide, L.pl.—*L. plantarum*, L.rh.—*L. rhamnosus*, un—untreated control.

The combined effect of PMs with TMZ and ARA12 on cell death processes in four different patient-derived GB cell lines (IDH-wildtype, verified additionally on cell cultures by real-time PCR for *IDH*1 R132H and *IDH*2 R172K codons) was examined by FC annexin V/PI assay. The results show the varied response of each GB cell line to the applied PMs and PM–drug combinations; however, no cytoprotective effect of PMs was detected in any of the examined neoplastic cell lines (Figure 6).

In the case of patient-derived GB cell lines, GBa and GBb have presented a significant (*p*< 0.05) increase in apoptosis in response to treatments with both *L. plantarum* and *L. rhamnosus* PMs combined with TMZ when compared to TMZ alone (21.64% for TMZ vs. 30.2% for *L. plantarum* with TMZ, *p* = 0.03, and 31.82% for *L. rhamnosus* with TMZ, *p* = 0.008, on GBa and 15.05% vs. 35.63%, *p* = 0.001, and 45.75%, *p* < 0.0001, respectively, for GBb). In GBb cells, the TMZ alone treatment was not potent enough to induce a statistically significant apoptosis increase, while PM addition increased apoptosis occurrence 2–3 times. Moreover, in GBa and GBb cells, when compared to the untreated control, *L. rhamnosus* PM-treated samples have shown an apoptotic cell population boost (9.74% in untreated sample vs. 24.18% for *L. rhamnosus* PM, *p* = 0.0003, on GBa and 7.03% vs. 23.73%, *p* = 0.009, respectively, on GBb). As for the GBc cell line, both PMs were able to induce apoptosis (16.42% for untreated cells vs. 28.03% for *L. plantarum* and 28.31% for *L. rhamnosus*, *p* = 0.01 for both); however, they were ineffective in combination with TMZ, causing no further increase in its proapoptotic activity. Although GBc cells were the only ones that did not show a significant apoptotic surge in response to ARA12, the addition of both PMs resulted in considerable inflation of the apoptotic cell population (25.64% for ARA12 vs. 54.59% and 56.87% for combined treatments, *p* < 0.0001). The GBd cells appeared to be irresponsive to either PMs or TMZ alone and combined treatments. ARA12 application brought about a substantial escalation of apoptotic processes regardless of PM addition (48.46% compared to 5% in untreated control, *p* < 0.0001).

There was a case of modestly increased necrosis on GBa cells treated with ARA12 and *L. rhamnosus* PM in comparison to samples treated with solely ARA12 (4.8% necrotic cells vs. 9.7% necrotic cells, *p* = 0.0077). Also all treatments comprising ARA12 on GBd cells caused a slight increase (not exceeding 6%) in the necrotic cell population compared to the untreated control (*p* < 0.006).

**Figure 7:**
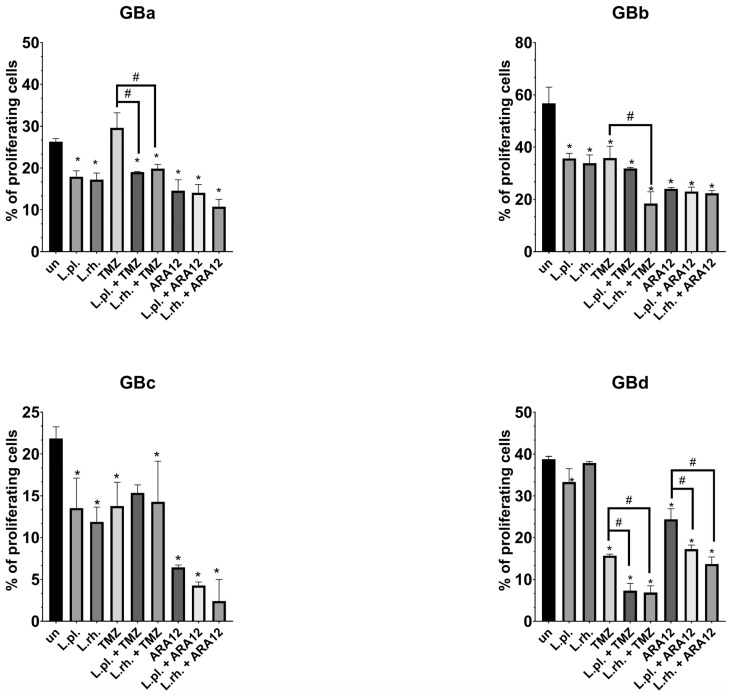
Drug and PM-induced GB cell line changes in proliferation rate. GBa, GBb, GBc, and GBd cell lines were treated with LAB-derived PM and their combinations with tested drugs for 72 h, then proliferation investigation based on EdU incorporation assay was conducted. Statistically significant differences between given sample and untreated cells are indicated with *, differences between samples are indicated with # (*p* < 0.05). TMZ—temozolomide, L.pl.—*L. plantarum*, L.rh.—*L. rhamnosus*, un—untreated control.

The cytostatic effect of PMs and their combinations with TMZ and ARA12 on different GB cell lines was examined with EdU-based proliferation assay. As in the case of previous experiments, the response to applied substances varied between cell lines. The results are shown in Figure 7. On GBb cells, PMs alone exerted an antiproliferative effect (56.77% EdU+ cells for untreated control vs. 35.68% for *L. plantarum* and 33.85% EdU+ cells for *L. rhamnosus*, *p* < 0.0001) and all treatment modes utilizing TMZ and ARA12 decreased the proliferation rate significantly; *p*< 0.05. GBa cell proliferation impediment was more pronounced in treatments combining TMZ and PMs than in TMZ alone (19.01% for *L. plantarum* + TMZ, *p* < 0.0001, and 19.85% for *L. rhamnosus* + TMZ (*p* = 0.0002) vs. 29.62% EdU+ cells for TMZ). GBa and GBc cells’ proliferation rate was also blocked by PMs alone (26.28% vs. 17.9% for *L. plantarum*, *p* = 0.001, and 17.2% EdU+ cells for *L. rhamnosus*, *p* = 0.0004, for GBa and 21.84% vs. 13.52% *for L. plantarum*, *p* = 0.017, and 11.89% EdU+ cells for *L. rhamnosus*, *p* = 0.004, for GBc). GBc cells were not sensitized by any of the PMs to drug-induced proliferation inhibition though. For GBd cells, *L. plantarum*-derived PM hindered the proliferation process (38.78% for untreated cells vs. 33.29% EdU+ cells for PM, *p* = 0.0071), and both PMs were able to significantly (*p* < 0.05) diminish the proliferation rate in combination with either of the applied drugs when compared to the drug alone treatment (for TMZ treatment: 15.7% vs. 7.34% for *L. plantarum* addition, *p* = 0.0001, and 6.85% EdU+ cells for *L. rhamnosus* addition, *p* < 0.0001; for ARA12 treatment: 24.42% vs. 17.29%, *p* = 0.0008, and 13.7% EdU+ cells, *p* < 0.0001, respectively).

### 3.4. Initial Screening of L. plantarum- and L. rhamnosus-Derived PM Activity Towards GB and Normal Cells Undergoing Irradiation Process Demonstrates More Evident Influence of PM in Maximal Tested Radiation Dose

**Figure 8:**
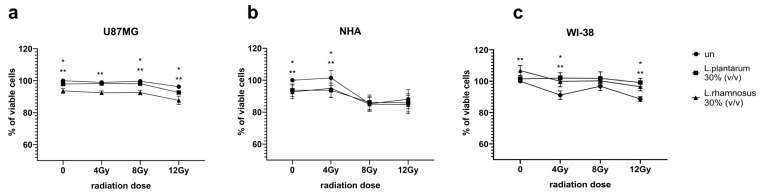
Radiation dose-dependent analysis of cells’ viability. U87MG (**a**), NHA (**b**), and WI-38 cells (**c**) were pretreated with 30% (*v*/*v*) PM. Viability was assessed by PrestoBlue assay 72 h after irradiation. Statistically significant differences between given sample treated with *L. plantarum* (*) or *L. rhamnosus* PM (**) and irradiated, untreated with PM; *p* < 0.05. un—control undergoing the same irradiation conditions but not pretreated with PMs.

In order to conduct an exploratory assessment of potential radiosensitizing properties of PMs towards GB cells, the U87MG (Figure 8a) cell line was utilized, while PMs’ radioprotective activity was examined on NHA (Figure 8b) and WI-38 cells (Figure 8c).

The results have shown a significant (*p* < 0.05) decrease in the viability of GB cells treated with *L. rhamnosus* PM in all applied radiation dosages, while a *L. plantarum* PM radio-sensitizing effect was not evident in 4 Gy radiation mode. Although the addition of PM slightly influences NHA viability prior to irradiation, it does not decrease it when subjected to 4 Gy, in relation to untreated sample. Additionally, 8 Gy appeared to be a dosage causing a significant decrease in astrocytes’ viability in relation to the control independently of PM addition; thus, PM does not provide a stronger negative effect on their response to 8 and 12 Gy; however, no cytoprotective activity was observed. On WI-38 cells, the effective cytoprotective action of *L. rhamnosus* PM and *L. plantarum* PM was observed in the case of maximal applied radiation dose 12 Gy.

### 3.5. Examined Postbiotics Modestly Enhance Apoptosis in Irradiated U87MG Cells and L. plantarum PMs Mitigate Pro-Senescent Irradiation Effect

**Figure 9:**
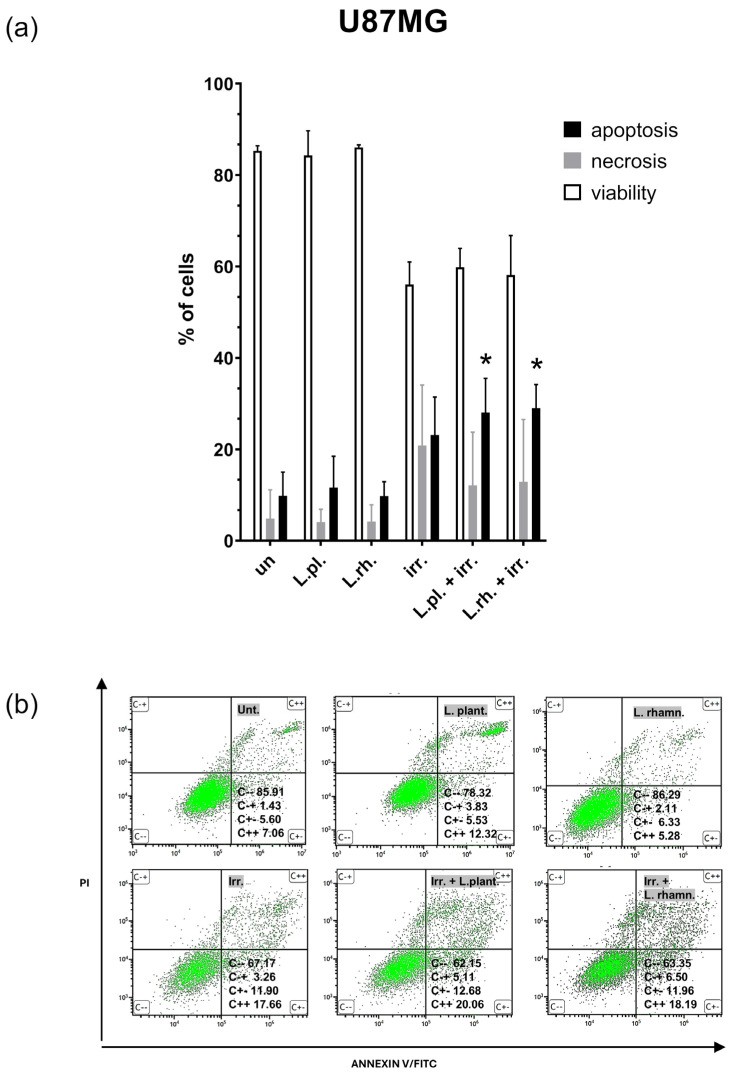
PMs and irradiation induced cell death processes on U87MG cells. (**a**) FC apoptosis assay (annexin V/PI staining)-based analysis of cell death processes in U87MG cells in response to 24 h pretreatment with PM and 12 Gy radiation conducted 72 h after irradiation. Statistically significant differences in apoptotic population size between given sample and untreated cells are indicated with * (*p* < 0.05). (**b**) the representative results of FC data, C − − viable cells, C − + necrotic cells, C + − early apoptotic cells, C + + late apoptotic cells. L.pl.—*L. plantarum*, L.rh.—*L. rhamnosus*, irr.—irradiation, un—untreated control.

The results of FC analysis of irradiated U87MG cells are shown in Figure 9. The only significantly enriched apoptotic cell populations when compared to the untreated control occurred in treatment modes combining PMs and irradiation (9.84% for untreated cells vs. 28.06% for irradiation and *L. plantarum* and 28.99% for *L. rhamnosus*, *p* < 0.05, the apoptotic population in solely irradiated sample was 23.11%). It is worth noting that combined treatment with PMs and radiation resulted in significant apoptosis increase in opposition to irradiation alone, without inducing necrosis.

**Figure 10:**
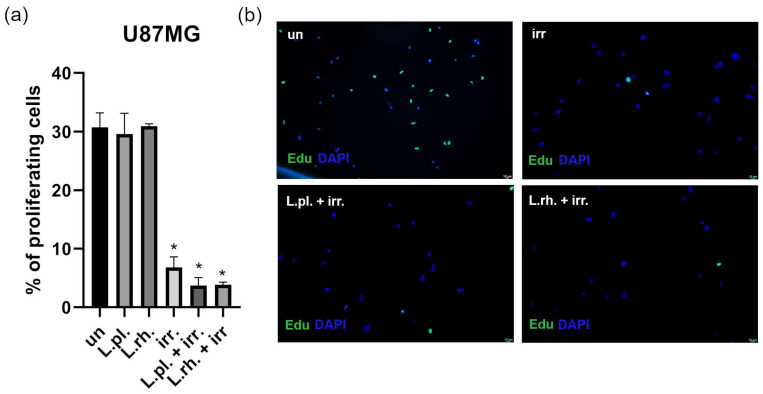
U87MG cells’ proliferation rate in response to 24 h pretreatment with LAB-derived PM and irradiation with 8 Gy. (**a**) Quantitative analysis of proliferation assay output (**b**) Representative results of EdU incorporation assay performed 72 h post irradiation procedure; Statistically significant differences between given sample and untreated cells are indicated with * (*p* < 0.05). L.pl.—*L. plantarum*, L.rh.—*L. rhamnosus*, irr.—irradiation, un—untreated control.

Proliferation assay results are presented in Figure 10. No significant cytostatic effect of examined PMs was observed on U87MG; however, all conditions comprising irradiation decreased the proliferation rate by more than 20%. Although not statistically significant, postbiotic pretreatment of irradiated cells lowered the proliferation rate by nearly half compared to irradiated cells that were not pretreated (6.82% of EdU+ cells for irradiation only vs. 3.7% and 3.85% of EdU+ cells for cells pretreated with *L. plantarum* and *L. rhamnosus* PMs, respectively). Importantly, the effect of irradiation in a single mode treatment was prominent; therefore, its intensification could prove implausible.

**Figure 11:**
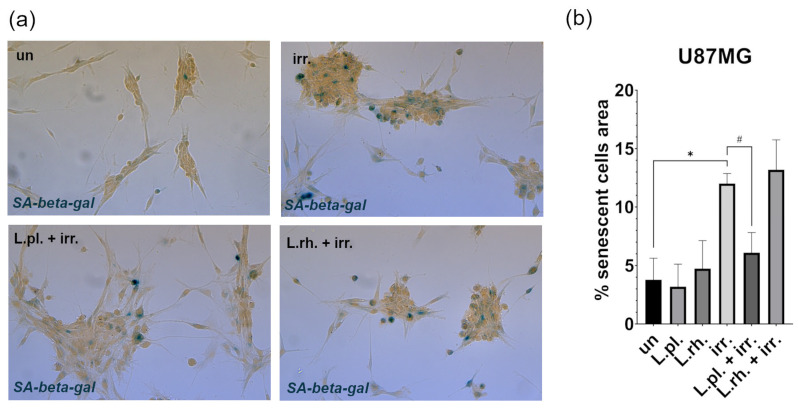
Senescence on U87MG cells in response to treatment with LAB-derived PM and their combinations with irradiation. (**a**) Representative results of SA-β-gal detection assay (20x magnification); (**b**) quantitative analysis of SA-β-gal test output. Statistically significant differences between given sample and untreated cells are indicated with *, differences between samples are indicated with #. Due to the specificity of cell growth, forming clusters, the values are expressed as percentage of the positive signal area to the area occupied by cells (*p* < 0.05). L.pl.—*L. plantarum*, L.rh.—*L. rhamnosus*, irr.—irradiation, un—untreated control, SA-β-gal—senescence-associated beta-galactosidase.

Although irradiation was proven to be a strong pro-senescent factor, the induced senescence intensity was mitigated by *L. plantarum*-derived PM (Figure 11). The irradiation procedure caused a significant intensification (3.78% vs. 12.01% of senescent cells area, *p* = 0.0001) of cellular senescence on U87MG cells. The effect was alleviated by *L. plantarum*-derived PM, decreasing the area occupied by senescent cells by nearly 6% (*p* = 0.004). This phenomenon was accompanied by a slight increase in apoptotic cells’ percentage (Figure 9).

### 3.6. The Effect of Pretreatment with L. plantarum- and L. rhamnosus-Derived PM in Combination with Irradiation on Cell Death Processes and Proliferation Rate Varies Between Examined GB Patient-Derived Cell Lines

**Figure 12:**
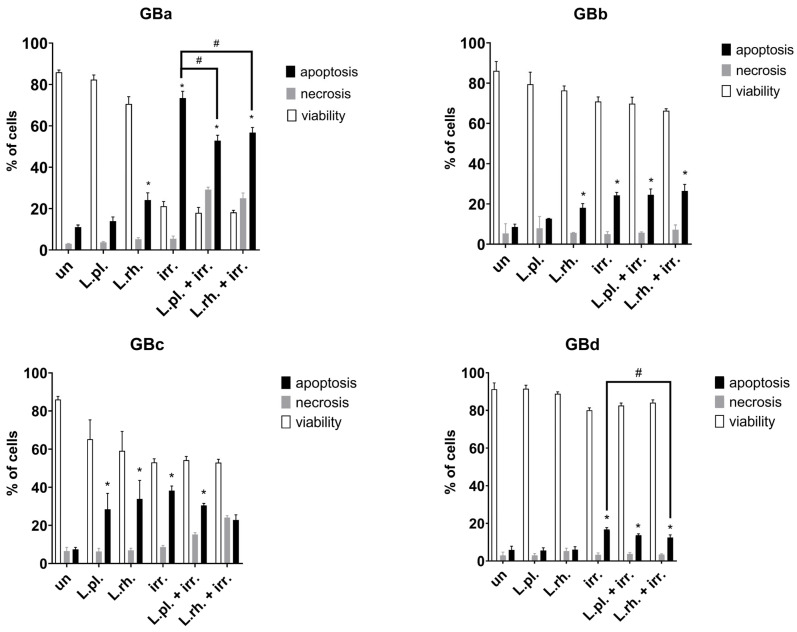
Cell death processes induced by PM treatment and irradiation in patient-derived GB cell lines. FC apoptosis assay (annexin V/PI staining)-based analysis of cell death processes in patient-derived GB cell lines, GBa, GBb, GBc, and GBd, in response to 24 h pretreatment with PM and 12 Gy radiation conducted 72 h after irradiation. Statistically significant differences assessed for percentage of apoptotic cells between given sample and untreated cells are indicated with *, differences between samples are indicated with # (*p* < 0.05). L.pl.—*L. plantarum*, L.rh.—*L. rhamnosus*, irr.—irradiation, un—untreated control.

Cell death process analysis by FC annexin V/PI assay, presented in Figure 12, did not show any significant radiosensitizing effect of PMs compared to irradiation alone on any of the examined cell lines in terms of increase in apoptotic cell population. Unfortunately, in GBa and GBc, pretreatment with PMs before irradiation resulted in increased necrosis (5.46% for irradiation alone vs. 29.21% for *L. plantarum* addition and 24.97% for *L. rhamnosus* pretreated cells, *p* < 0.0001, in GBa and 8.66% vs. 15.25%, *p* = 0.0003, and 24.15%, *p* < 0.0001 in GBc, respectively) and a decrease in apoptotic cell population. In GBb cells, PMs did not significantly affect irradiation effects. In the case of the GBd cell line, the *L. rhamnosus* PM alone remained ineffectual, and it exerted a slight antiapoptotic effect on irradiated GBd cells (16.68% for irradiation vs. 12.46% for PM pretreatment, *p* = 0.02), while *L. plantarum* PM alone had no impact on the cells, nor did it interfere with irradiation outcomes.

**Figure 13:**
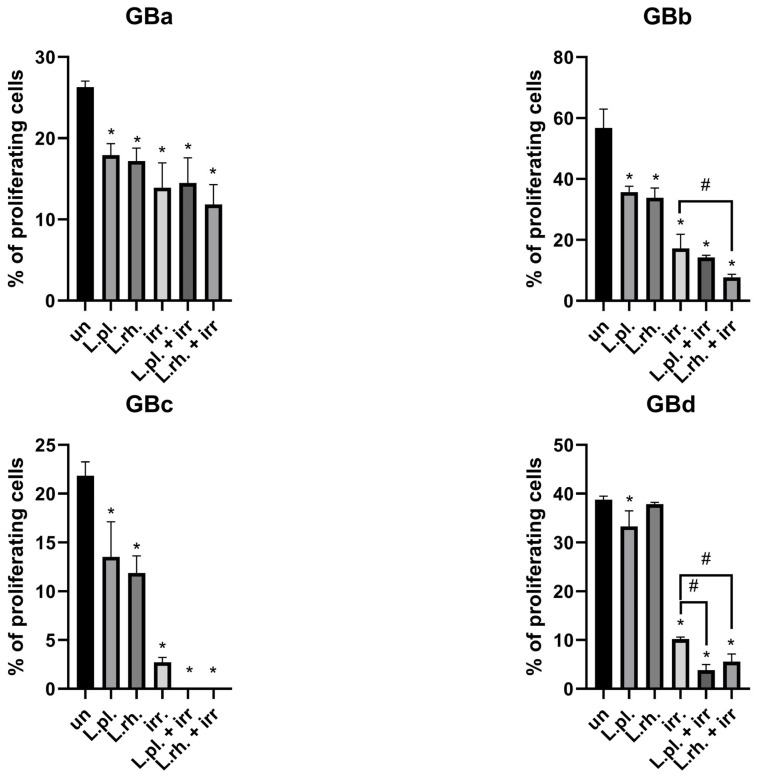
Proliferation rate changes in patient-derived GB cell lines after PM treatment and irradiation. EdU incorporation assay-based assessment of patient-derived GB cell lines, GBa, GBb, GBc and GBd, with changes in proliferation rate in response to 24 h pretreatment with LAB-derived PM and irradiation with 8 Gy, which were then incubated for 72 h. Statistically significant differences between given sample and untreated cells are indicated with *, differences between samples are indicated with # (*p* < 0.05). L.pl.—*L. plantarum*, L.rh.—*L. rhamnosus*, irr.—irradiation, un—untreated control.

The proliferation rate of 8 Gy irradiated GB cells, assessed with EdU incorporation assay (Figure 13), was affected to a variable degree by *L. plantarum* and *L. rhamnosus* PM pretreatment, depending on the studied cell line. On GBa cells, no enhanced cytostatic effect was observed. On GBc cells, no significant effect of PMs was detected when compared to irradiation alone; however, in the treatment modes entailing PM pretreatment and subsequent irradiation, no proliferating cells were identified (vs. 2.72% proliferating cells in solely irradiated sample). In GBb and GBd cells, the *L. rhamnosus*-derived PM further diminished the proliferation rate when compared to irradiation alone (17.23% of EdU+ cells in irradiation alone mode vs. 7.67% for *L. rhamnosus* PM pretreatment, *p* = 0.02, on GBb and 10.19% vs. 5.57% of EdU+ cells, *p* = 0.002, on GBd, respectively). The cytostatic effect was also observed on GBd cells pretreated with *L. plantarum*-derived PM (10.19% for irradiation alone vs. 3.8% of EdU+ cells for PM pretreatment, *p* = 0.01).

### 3.7. Short Summary of Postbiotic Effect on Examined Processes in Patient-Derived GB Cells

In order to conveniently present and summarize the obtained outcomes, the results of combinatory treatments applied to patient-derived GB cells are summarized in Table 1.

## 4. Discussion

Despite tremendous scientific efforts aimed at glioblastoma’s perplexing ability to escape numerous therapeutic attempts, the concern remains unsolved. Our experimental approach addressed the obstacles hindering tumor response, such as treatment-induced resistance and GB heterogeneity, as well as a possibility to boost the effectiveness of oncotherapy without causing harm to normal cells.

We have observed postbiotics modulating the tumor cell response to treatment, which could minimize the risk of therapy escape. The oncological treatment induces several cellular processes responsible for the final effect, among which the most desired one is proliferation inhibition and cell death stimulation without excessive necrosis. Cellular senescence, a mechanism previously favorably seen in the course of anti-neoplastic therapies, has recently been proven to hinder therapy effectiveness, increase the risk of tumor relapse, and contribute to undesired side effect occurrence via its proinflammatory activity [11,31,32]. Senescence induced by chemo- and radiotherapy applied to combat glioblastoma has not only been identified as one of the tumor therapy escape mechanisms, but was also observed to affect non-neoplastic CNS cells, which, through SASP, further aggravate deleterious tumors’ microenvironment and promote glioblastoma growth [11,33,34]. In light of the recent findings, postbiotics have been recognized as natural agents with anti-inflammatory properties and the potential to mitigate senescence-driving processes [35,36]. Additionally, they presented selective cytotoxicity against cancer cells, making them promising tools to support standard oncotreatment regimens [14,17]. In the current study, we have examined postbiotics’ influence on TMZ, ARA12, and irradiation-induced cellular senescence on the GB cell line U87MG. As suspected, both TMZ and the drug candidate ARA12 caused a significant intensification of senescence processes compared to the untreated control sample. In the TMZ condition, both PMs lowered senescence occurrence, while in the ARA12 treatment, only the *L. plantarum* PM was effective. Importantly, the *L. plantarum* PM sensitized GB cells to the proapoptotic effect of both drugs, significantly increasing the apoptotic cell population also in comparison to the only drug-treated samples, and *L. rhamnosus* PM acted so in combination with ARA12. The irradiation procedure also provoked a prominent increase in senescence. *L. plantarum* PM pretreatment ameliorated the pro-senescent effect of radiation. Both PMs sensitized U87MG cells to proapoptotic irradiation activity without triggering more prominent necrosis.

Although not affected by PMs alone, proliferation was significantly inhibited by both tested drugs and their combinations with PMs. *L. rhamnosus* PM addition to TMZ enhanced drug’s antiproliferative activity. Irradiation treatment alone caused a severe decrease in the proliferation rate and no statistically significant enhancement of this treatment was observed with PM addition, although the trend was sketched.

Previous reports show therapeutic benefits of the addition of senolytics or senomorphics to increase apoptosis by targeting treatment-induced senescence cells [17,37,38,39]. Our results suggest that supplementing both TMZ and ARA12 with postbiotics has the potency to shift tumors’ therapeutic response towards apoptosis by decreasing treatment-induced senescence.

However, our outcomes regarding the response to PM in combinatory treatment modes were not unified across patient-derived GB cell lines (Table 1). GB are known for their heterogenic nature reflected by varying sensitivity to treatment [4,40]. To determine putative differences in response to applied treatment, the current project includes four patient-derived GB cell lines. Published results of research regarding postbiotics’ influence on patient-derived material, rather than commercially available cell lines, are still scarce.

In the current study, both examined postbiotic mixtures, obtained from *L. plantarum* or *L. rhamnosus*, exert cytotoxic or cytostatic effects on all examined GB cell lines and do not increase necrosis when applied alone. The *L. plantarum* PM boosted apoptosis on one patient-derived cell line, while the *L. rhamnosus* PM had the same effect on three tested patient-derived cell lines. Three patient-derived GB cell lines responded to either PM alone with proliferation decreasing when compared to untreated samples.

When applied with TMZ, PMs show potential to increase treatment effectiveness detected as apoptosis enhancement or proliferation block; however, the boosting effect varied between patient-derived cell lines and was observed in three of them (except for GBc). The addition of *L. plantarum* or *L. rhamnosus* PM sensitized some initially resistant GB cell lines to the proapoptotic (GBb) or cytostatic (Gba) activity of the TMZ.

ARA12, the drug candidate, has proven to be more effective than TMZ in triggering apoptosis, as only one patient-derived cell line (GBc) was unresponsive to its proapoptotic action, but, as in the case of U87MG, both PMs sensitized it to the treatment. All ARA12 treatment modes caused a proliferation decrease compared to untreated cells on all four tested cell lines. An enhanced cytostatic effect of combination treatment for the drug candidate was observed on one cell line (GBd) for either PM addition. Importantly, the enhanced effect of ARA12 may be difficult to observe, since the drug alone causes a prominent decrease in cells viability.

A significant decrease in GB cells’ viability compared to untreated samples was present in all conditions comprising irradiation on all examined cell lines. In contrary to experiments with chemotherapeutic agents, the addition of PM to the irradiation regime did not result in apoptosis enhancement; however, considering the viability of GB cells, no radioprotective activity of PM addition was observed. Considering that excessive necrosis is an unwanted therapy effect promoting immunogenic and proinflammatory pathways of cellular death, we aimed to verify if treatment regimens combined with PM decrease the rates of necrotic cell population compared to single modes of treatment. Although some ARA12-treated samples presented the occurrence of necrotic cells, the necrotic population in combined treatment modes was no more significant than that of the drug-alone mode and did not exceed 10%, while the number of apoptotic cells was in the range of 30–50%, depending on the GB line and treatment condition. The pro-necrotic action of irradiation was unexpectedly enhanced in the case of two cell lines when pretreated with both PMs, which resulted in an unfavorable change in the proportion of necrotic and apoptotic cell populations. However, the pro-apoptotic potential of these treatments remained significant compared to untreated samples. Moreover, though pretreatment with PMs prior to irradiation gave no promising results in the context of cell death stimulation on patient-derived GB cells, their action gives more unequivocal output in enhancing proliferation inhibition.

Besides boosting the therapeutic action to fight neoplastic cells, cancer treatments would benefit from including selectively a cytoprotective agent that would protect normal cells. Agents with such potential, still waiting to be fully elucidated, are postbiotics. Elderly cancer patients undergoing invasive treatments are unfortunately increasing in number, which is not predicted to change in the near future. The number of cancer survivors who still suffer cognitive deficits caused by oncotherapy is also growing. However, the research regarding cognitive decline caused by the damage done to normal cells of CNS during chemo- and radiotherapy treatment is not conclusive. Our results demonstrate that TMZ has slight cytotoxicity to NHA, but also to most GB cell lines examined in the study, showing its modest effectiveness as a standard anti-neoplastic drug. The application of chemicals dealing more damage to neoplastic cells, but presenting limited selectivity, such as ARA12, might require the employment of an agent with cytoprotective activity toward normal cells. The PMs had a beneficial influence on fibroblasts’ viability and despite slight cytotoxicity to astrocytes when applied alone, they significantly rescued their viability when treated with the novel candidate therapeutic—ARA12. ARA12 had previously been proven to exert a greater proapoptotic effect on GB cells than routinely applied TMZ, when applied at higher concentrations [13]. However, the drug candidate was harmful towards normal astrocytes; thus, a lower dosage of this new compound was examined in the current study. ARA12, even applied at a lower concentration of 50 μg/mL, affected the viability of more GB cell lines than TMZ, and combinations of ARA12 with PM significantly enhanced its cytotoxicity, resulting in apoptosis occurrence. Moreover, the addition of PMs diminished the damage to examined normal cell lines, which could lessen chemotherapy’s adverse side effects. The results demonstrate that both examined PMs, obtained from *L. plantarum* or *L. rhamnosus*, exert cytotoxic or cytostatic effects on all examined GB cell lines, while maintaining neutral or positive influence on normal NHA and WI-38 cells when applied alone.

Clinical trials utilizing postbiotics have proven their beneficial effect on CNS cells—for instance, lessening neuroinflammation in patients recovering from stroke and improving overall mood in healthy adults consuming probiotic-fermented milk, as well as on liver cells endangered by liver fibrosis [41,42,43]. Postbiotics’ application in ameliorating senescence in the CNS has mostly been analyzed in neurodegenerative diseases [18].

The dual activity of postbiotics presenting both anti-neoplastic and cytoprotective potential has been verified not only in vitro, but also as output of in vivo studies. Postbiotics have been examined as agents supporting a anti-neoplastic effect of routinely applied drugs, for instance, butyrate supplementation was observed to support the chemotherapeutic activity of gemcitabine in pancreatic ductal adenocarcinoma in vitro and on a mouse model and also lowered markers of liver and kidneys damage [38]. *Weizmannia coagulans* MZY531 postbiotics ameliorated CT-26 colorectal tumor growth in mice by regulating apoptotic and autophagic pathways [37].

The phenomena of intra-tumor and inter-tumoral heterogeneity and GB plasticity are considered to be among the most important reasons for resistance and therapeutic escape [4,40,41]. Although the overall output of our study demonstrates the anti-neoplastic potential of postbiotics against GB, the involvement of four patient-derived tumors revealed different responses to the applied treatment in vitro. Despite significant efforts to identify correlations between the molecular and phenotypic profiles of GBs and their susceptibility to treatment, only the *IDH* mutational status has currently been included in the WHO diagnostic criteria as a clinically relevant factor, showing that IDH-wildtype tumors are more resistant to therapy [44,45]. The available literature shows that the molecular status of GB genetic hallmarks, as well as the methylation status and transcriptomic subtypes classified as proneural, neural, classical, and mesenchymal, also influence GB treatment responsiveness. However, more advanced analyses of GB transcriptomes in large populations appear to suggest a more nuanced explanation for variability in treatment response, which hinders the design of personalized therapy [4,29,30].

The promising results regarding postbiotics bring hope to break the therapeutic impasse in combating glioblastoma, occurring partially due to the unmutated *IDH* gene further hindering treatment of the incurable tumor. The proper activity of IDH enzymes, converting isocitrate to alpha-ketoglutarate in IDH-wildtype cells, allows for maintaining the demethylation of DNA and histones, as well as cells’ undisturbed metabolic functioning, which is appointed as one of the factors contributing to GB therapy resistance [41]. Moreover, *IDH*-mutated gliomas, such as astrocytoma, may be more susceptible to chemo- and radiotherapies due to impaired DNA repair mechanisms and metabolic stress and there is a possibility of utilizing small molecules, such as ivosidenib or vorasidenib, to inhibit mutated *IDH*, whereas inhibition is not possible for IDH-wildtype GB [46,47]. Therefore, the search for therapeutics that could improve the protocol for combating GB is still very much active and needed.

The aim of our experimental approach, which employed a few patient-derived GB cultures, was to sketch the diversity of GB’s responsiveness to postbiotic treatment. However, at this stage, searching for links between their molecular/phenotypic profiles and susceptibility to the applied regimens is limited by the quantity of samples. Furthermore, LAB-derived postbiotics are mixtures of several compounds, meaning that the way they affect the cells is the result of their bioactivity and influence on pathways involved in cell survival and death. Since most reports show the promising anti-neoplastic potential of postbiotics based exclusively on commercial cell lines, our results emphasize the importance of using patient-derived models to verify variability in effects.

## 5. Conclusions

In our work, both postbiotic mixtures exerted treatment-supporting effects to a similar degree, sensitizing GB cells to apoptotic and antiproliferative treatments’ effects; nonetheless, their influence on radiotherapy effectiveness remains incongruent, as they sensitize one GB cell line to the therapeutic effect while partially hindering the treatment on other cell lines. The *L. plantarum*-derived PM appeared to be more effective in its anti-senescent activity in both chemo- and radiotherapy modes, producing very promising results for combating senescence-associated therapy escape. However, the relationship between postbiotics and treatment-induced senescence in glioblastoma needs further analyses. The obtained results support the thesis that postbiotic-based adjunctive treatment could potentiate the therapeutic action toward neoplastic cells, while alleviating chemotherapy’s adverse effects, to tackle the issue of therapy resistance and improve patients’ comfort. Importantly, the output yielded from patient-derived GB cell lines suggests that in future research, special attention should be paid to the heterogeneity of tumor response to treatment, a phenomenon often neglected in reports showing promising effects of postbiotics as oncotherapy adjuncts.

## Figures and Tables

**Table 1:** Comparison of results obtained for examined cellular processes on patient-derived cell lines.

Cell Line	Treatment Mode		Cellular Processes		
			Apoptosis Stimulation	Necrosis Occurrence	Proliferation Inhibition
GBa	*L. plantarum* PM		0	0	+
	*L. rhamnosus* PM		+	0	+
	No postbiotic	TMZ	+	0	0
		ARA12	+	0	+
		irradiation	+	0	+
	*L. plantarum* PM	TMZ	+ enh	0	+ enh
		ARA12	+	0	+
		irradiation	+ hin	+ hin	+
	*L. rhamnosus* PM	TMZ	+ enh	0	+ enh
		ARA12	+	+ hin	+
		irradiation	+ hin	+ hin	+
GBb	*L. plantarum* PM		0	0	+
	*L. rhamnosus* PM		+	0	+
	No postbiotic	TMZ	0	0	+
		ARA12	+	0	+
		irradiation	+	0	+
	*L. plantarum* PM	TMZ	+ enh	0	+
		ARA12	+	0	+
		irradiation	+	0	+
	*L. rhamnosus* PM	TMZ	+ enh	0	+ enh
		ARA12	+	0	+
		irradiation	+	0	+ enh
GBc	*L. plantarum* PM		+	0	+
	*L. rhamnosus* PM		+	0	+
	No postbiotic	TMZ	+	0	+
		ARA12	0	0	+
		irradiation	+	0	+
	*L. plantarum* PM	TMZ	0	+	0
		ARA12	+ enh	0	+
		irradiation	+	+ hin	+
	*L. rhamnosus* PM	TMZ	0	0	+
		ARA12	+ enh	0	+
		irradiation	0	+ hin	+
GBd	*L. plantarum* PM		0	0	+
	*L. rhamnosus* PM		0	0	0
	No postbiotic	TMZ	0	0	+
		ARA12	+	+	+
		irradiation	+	0	+
	*L. plantarum* PM	TMZ	0	0	+ enh
		ARA12	+	+	+ enh
		irradiation	+	0	+ enh
	*L. rhamnosus* PM	TMZ	0	0	+ enh
		ARA12	+	+	+ enh
		irradiation	+ hin	0	+ enh

+—an increase in process intensity compared to untreated control; 0—no significant change in process intensity compared to untreated control; enh—an enhanced action of PM and therapeutic in treatment mode comprising PM and drug or irradiation compared to drug or irradiation applied alone; hin—PM hindering therapeutic effect in treatment mode comprising PM and drug or irradiation compared to drug or irradiation applied alone (*p* < 0.05).

## Data Availability

Dataset available on request from the authors.

## Abbreviations

The following abbreviations are used in this manuscript:

CNS	central nervous system
EdU+	EdU-positive cells
FC	flow cytometry
GB	glioblastoma
*IDH*	isocitrate dehydrogenase
Irr.	irradiated
LAB	Lactic acid bacteria
*L. plantarum*; *L.pl*	*Lactiplantibacillus plantarum*
*L. rhamnosus*; *L.rh*	*Lacticaseibacillus rhamnosus*
NHA	normal human astrocytes
PI	propidium iodide
PM	postbiotic mixture
SASP	senescence-associated secretory phenotype
SCFA	short chain fatty acid
TMZ	temozolomide
un	untreated
